# Effects of Combined Intervention of rTMS and Neurotransmitter Drugs on the Brain Functional Networks in Patients with Cognitive Impairment

**DOI:** 10.3390/brainsci13030419

**Published:** 2023-02-28

**Authors:** Mengyun Li, Zhiming Qin, Haifeng Chen, Zhiyuan Yang, Lianlian Wang, Ruomeng Qin, Hui Zhao, Feng Bai

**Affiliations:** 1 Nanjing Drum Tower Hospital Clinical College of Nanjing University of Chinese Medicine, Nanjing 210008, China; 2 Department of Neurology, Nanjing Drum Tower Hospital of The Affiliated Hospital of Nanjing University Medical School, and The State Key Laboratory of Pharmaceutical Biotechnology, Institute of Brain Science, Nanjing University, Nanjing 210008, China; 3 Nanjing Drum Tower Hospital Clinical College of Jangsu University, Nanjing 210008, China; 4 Geriatric Medicine Center, Affiliated Taikang Xianlin Drum Tower Hospital, Medical School of Nanjing University, Nanjing 210008, China

**Keywords:** cognitive impairment, neurotransmitters, rTMS, brain networks, functional connectivity

## Abstract

Alzheimer’s disease (AD) causes extensive neural network dysfunction. Memantine and donepezil are commonly used as monotherapy or in combination with non-drug interventions, such as repetitive transcranial magnetic stimulation (rTMS), for its treatment. However, no studies have reported any differences between the effects of combined neurotransmitter and rTMS interventions versus rTMS alone on the brain networks of patients with cognitive impairment. Therefore, it is crucial to explore the advantages of different intervention methods to guide clinical practice. We used resting-state functional magnetic resonance imaging (rs-fMRI) to investigate the impact of neurotransmitter superimposed rTMS and rTMS alone on the brain functional network of patients with cognitive impairment. We divided patients with cognitive impairment who had received rTMS into two groups based on whether they received neurotransmitters: the combined intervention group and the rTMS-alone intervention group. We conducted rs-fMRI scans and comprehensively assessed cognitive function in these patients. To examine the effects of the superimposed interventions, we utilized independent component analysis to evaluate the functional connectivity of brain networks in these patients. Compared to the rTMS-alone intervention group, co-intervention of neurotransmitter drugs and rTMS exhibited potential for cognitive enhancement via the reconstructed inter-network connectivity of the cerebellum and the enhanced intra-network connectivity of the frontal-parietal regions in these patients with cognitive impairment. We hypothesized that the combination of neurotransmitter drugs and rTMS intervention could have greater clinical benefits than rTMS intervention alone, leading to improved cognitive function in patients with cognitive impairment.

## 1. Introduction

Normal aging and neurodegenerative diseases lead to changes in brain structure and function, and decreased strength of functional network connections which are closely related to cognitive decline. Alzheimer’s disease (AD) manifested as cognitive dysfunction such as memory, executive function, and visual space function which is a typical representative of degenerative diseases. There is already evidence that AD is a complex polygenic disorder that is the result of multiple factors acting abnormally [1,2,3].

Although the pathogenesis of AD is not fully understood, current theories have guided the exploration of clinical interventions to delay the progression of AD. At present, the drugs used clinically mainly include acetylcholinesterase inhibitors (donepezil, etc.) and NMDA receptor antagonists (memantine). Acetylcholinesterase inhibitors (CHEIs) prevent acetylcholine from being destroyed in patients by blocking acetylcholinesterase activity, compensating for acetylcholine in the brain, thereby improving cognitive function [4]. Memantine is an N-methyl-D-aspartate (NMDA) receptor antagonist that non-competitively blocks NMDA receptors, reduces glutamate-induced NMDA receptor overexcitation, prevents apoptosis, and is effective in improving memory in patients with AD [5].

Prior research has indicated that administration of donepezil to patients with mild cognitive impairment (MCI) for a duration of 3 to 6 months may result in enhanced brain activation during memory processing in the left inferior frontal gyrus, a region linked to attention and memory processes such as encoding and retrieving short- and long-term memory [6]. Additional research demonstrated that patients with MCI who received donepezil for 3 months displayed increased activation in the right medial temporal lobe, including the hippocampus/parahippocampal gyrus, during episodic memory coding tasks. Increased functional connectivity was significantly linked to improved fMRI task performance. Results suggest that donepezil may partially “normalize” brain activation patterns and connections during tasks, and these effects are linked to cognitive changes [7]. Furthermore, there is growing evidence that memantine treatment can have a beneficial effect in patients with moderate to severe Alzheimer’s disease (AD), as it can lead to increased resting default mode network (DMN) activity in the precuneus region over 6 months [8]. Therefore, the drugs may improve cognitive function by improving functional connectivity of brain networks.

Non-drug interventions have been shown to have important implications for slowing the progression of Alzheimer’s disease (AD) and improving cognitive function. Repeated transcranial magnetic stimulation (rTMS) has been shown as one of the effective strategies to improve cognitive function in patients with cognitive impairment [9]. Previous studies have demonstrated that repetitive transcranial magnetic stimulation (rTMS) directly stimulates cortical neurons by generating magnetic fields that induce electrical currents and activate the synaptic activities of neuronal circuits in the central nervous system [10]. Moreover, an rTMS study located in the left angular gyrus demonstrated that one month of treatment with rTMS was effective in improving patient episodic memory [3]. It was reported that rTMS can also affect cognitive function by affecting functional connectivity of brain networks. For example, studies have shown that rTMS modulation directed upon the precuneus for 2 weeks could improve middle cognitive region (HIPc) connectivity with the left parahippocampal gyrus and posterior perceptual region (HIPp) connectivity with the left middle temporal gyrus, potentially improving episodic memory [11]. Moreover, a study conducted by Wang et al. (2014) demonstrated that repetitive transcranial magnetic stimulation (rTMS) administered to the posterior parietal cortex (PPC) five times daily resulted in a significant increase in functional connectivity within hippocampus-centric networks and an improvement in cognitive function [12,13]. As an effective non-invasive intervention that can be combined with drugs for the treatment of patients with clinical cognitive impairment, this study was designed to explore the differences between the effect of neurotransmitter-drug-superimposed rTMS treatment and rTMS treatment alone on the functional connectivity of patients’ brain networks.

Independent component analysis (ICA) is an effective tool for separating statistical independent source signals in blood oxygen level dependent (BOLD) data. In the absence of a prior seed region, the separation of different functional magnetic resonance (fMRI) signal sources by maximizing the non-Gaussian nature of the source signal can identify intracranial function during rest. These networks are often referred to as “intrinsically connected networks” (ICNs) or “rest state networks” (RSNs) [14,15]. This study attempted to explore: (i) how neurotransmitter-superimposed rTMS interventions affect the functional connections of brain networks; and (ii) whether the changes in the functional connections of brain networks were associated with behavior performance in these patients. This study will provide a deeper understanding of the impact of combined use of interventions on brain functional networks in patients with cognitive impairments.

## 2. Materials and Methods

### 2.1. Participants

This study was approved by the Ethics Committee of Nanjing Drum Tower Hospital and the written informed consent of all patients was obtained before entering the study. In this study, patients with cognitive impairment who had received rTMS treatment were screened in the Department of Neurology, Drum Tower Hospital, Nanjing University School of Medicine. Four of the patients were excluded due to excessive head movement (>3 degrees) during fMRI scans, and two patients were excluded due to incomplete behavioral data. As shown in Table 1, the study enrolled 28 patients with cognitive impairment (all Chinese Han and right-handed), including 11 males and 17 females. These participants included patients with MCI and AD, and the diagnostic criteria for patients with AD were based on standards developed by the National Neurological and Communication Disorders and Stroke Institute and the Association for AD-Related Diseases (NINCDS-ADRDA Criteria) [16], while combining cerebrospinal fluid pathology and neuroimaging student markers for judgment. Patients with MCI were diagnosed based on criteria established by the previous studies [17,18,19,20,21,22]. According to research conducted by May and colleagues (Petersen et al., 1999, published in the *Archives of Neurology*), the diagnostic criteria for Alzheimer’s disease include: (i) a memory complaint, preferably confirmed by an informant; (ii) objective evidence of memory impairment for the individual’s age; (iii) generally preserved cognitive function for the individual’s age; (iv) activities of daily living that are essentially intact; and (v) the individual is not considered to be demented. All participant exclusion criteria included: (i) a history of neurological or psychiatric disorders (brain tumors, epilepsy, Parkinson’s disease, severe anxiety and depression, thyroid dysfunction, or other neurological or psychiatric disorders that may contribute to memory loss); (ii) any MRI contraindications or poor image quality; (iii) all neuropsychological assessments could not be completed.

### 2.2. Repetitive Transcranial Magnetic Stimulation Intervention

Our intervention targets are provided by another study in our research group, who used the left hippocampus as the seed, calculated by seed-based functional connectivity analysis, and ultimately located the intervention target in the left corner gyrus (MNI: −45, −67, 38). rTMS was delivered using a commercially available magnetic stimulator (CCY-IV model; YIRUIDE Inc., Wuhan, China) with a 70 mm figure-eight coil and an electromyography device. Each stimulation session consisted of forty circulations of 2 s delivered at 20 Hz spaced by 28 s of no stimulation, for a grand total of 1600 stimulations. The stimulation target is defined as a sphere of 6 mm radius centered at MNI coordinates (MNI: −45, −67, 38) and the treatments lasted about 20 min. For detailed information, please refer to the study of Yang 2022, published in *J Alzheimers Dis* [3]. Participants in this study were all treated with rTMS for a period of four weeks, followed by an fMRI scan and a complete neuropsychological assessment. At the same time, statistics of patients taking neurotransmitter drugs were captured. To the best of our knowledge, patients were administered a daily dose of 10 mg of donepezil or 10 mg of memantine, and the average duration of medication was more than eight weeks. The neurotransmitters refer to the AChEIs (e.g., donepezil) and NMDA receptor antagonist (e.g., memantine).

### 2.3. Neuropsychological Assessment

To assess the behavioral effects of the treatment, we employed a standardized neuropsychological test protocol that includes global cognitive assessment and multiple cognitive domain examinations. We also completed the Clinical Dementia Rating scale to assess the extent of cognitive impairment in participants. We used the Activity of Daily Living Scale to assess the patients’ ability to perform activities of daily living. Global cognitive function was assessed by MMSE and Montreal Cognitive Assessment Beijing (MoCA-BJ). The raw test scores were converted to Z-scores, which were used to calculate the compound cognitive index. Episodic memory was calculated as the average of the Z-scores from the AVLT-DR scores and the Webster Memory Scale-Visual Reproduction-delayed Recall (VR-DR). AVLT-DR was determined based on the number of words retrieved 20 min after learning trials of 15 words, and VR-DR assessed ability to reproduce difficult to verbalize designs after a brief exposure. The information processing speed was calculated as the average Z-scores of the Trail Making Test-A (TMT-A) and the Stroop Color and Word Tests A and B (Stroop A and B). Language function included the Boston Naming Test and the Category Verbal Fluency test. The execution function was a composite score of the average Z-scores of the digital Span Test-backward, Trail Making Test-B (TMT-B), and Stroop Color and Word tests C (Stroop C). The visual spatial function was the average of a composite score that includes the Z-scores of the Clock Drawing Test and the Visual Replication-copy Test [3].

### 2.4. fMRI Acquisition

All participants were examined on a Philips 3.0T scanner (Philips Medical System). The inspection protocol included a high-resolution T1-weighted turbine gradient echo sequence (repeat time [TR] = 9.8 ms, flip angle [FA] = 8°, echo time [TE] = 4.6 ms, FOV = 250 × 250 mm^2^, number of slices = 192, acquisition Matrix = 256 × 256, thickness = 1.0 mm), the FLAIR Sequence (TR = 4.500 ms, TE = 333 ms, Time Interval [TI] = 1.600 ms, number of slices = 200, voxel size = 0.95 × 0.95 × 0.95 mm^3^, acquisition matrix = 270 × 260) and the resting-state function scans imaging sequence (TR = 2000 ms, TE = 30 ms, FA = 90, acquisition matrix = 64 × 64, number of slices = 35, thickness = 4.0 mm, FOV = 240 × 240 mm^2^) [3].

### 2.5. Data Preprocessing

We used statistical parametric mapping (SPM)-12 software (http://www.fil.ion.ucl.ac.uk/spm) and the Resting State fMRI Data Processing Assistant (http://rfmri.org/DPARSF) to preprocess resting BOLD data [23]. The following steps were adopted: (i) Converting the data from the medical digital imaging format to the NIfTI format. (ii) The first 10 volumes were discarded to allow the signal to reach equilibrium and the participants to adapt to the environment, leaving 220 images for further processing. (iii) The remaining 220 time points were corrected for the acquisition time delay between different slices, ensuring that all voxels in a volume were scanned instantaneously at the same moment. (iv) The rigid-body head movement during scans was corrected. Excessive motion was defined as more than 3.0 mm of translation or greater than a 3.0° rotation in any direction and two patients were excluded; thus, 30 subjects were included for further analysis. (v) The functional images were then spatially normalized to the Montreal Neurological Institute (MNI) space using a unified segmentation algorithm. Then, the data were resampled to an isotropic resolution of 3 mm using the parameters estimated during unified segmentation [24]. (vi) Spatial smoothing: the normalized images were spatially smoothed using an isotropic Gaussian filter with a full width half maximum (FWHM) of 6 mm to reduce spatial noises [25,26,27].

### 2.6. Identification of Resting-State Networks

**Figure 1:**
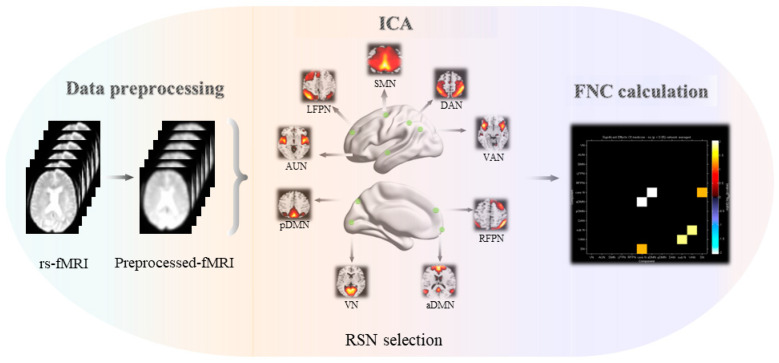
General steps of functional network connectivity analysis. A total of 34 ICs were obtained by GICA, then 34 ICs were assigned to twelve brain networks. Abbreviations: ICs, independent components; GICA, group independent component analysis.

We used the GIFTv4.0 software (ICA in the fMRI toolbox, http://icatb.sourceforge.net, 26 February 2023) to analyze the preprocessed images, following three main steps: (i) data reduction, (ii) group ICA, and (iii) back reconstruction. First, principal components analysis (PCA) was applied to reduce the data dimensionality for each subject. The reduced data from all subjects were then concatenated and entered into a second data reduction step using PCA. Second, the simplified data were then estimated using the Informax algorithm to estimate the spatially independent components (34 in this study), and Informax was run 20 times in the ICASSO toolbox algorithms to increase the reliability of independent component decomposition. Finally, individual subject components were back reconstructed from the group components using the GICA approach, during which the aggregate components and the results from the data reduction step were used to compute the individual subject components. Each back-reconstructed component consists of a spatial z-map reflecting the component’s functional connectivity pattern across space and an associated time course reflecting the component’s activity across time [2,15,28]. The group-level components corresponding to the visual network (VN), the auditory network (AUN), the sensorimotor network (SMN), the left frontoparietal network (LFPN), the right frontoparietal network (RFPN), the cerebellum network (CN), the anterior default mode network (aDMN), the posterior DMN (pDMN), the dorsal attention network (DAN), the ventral attentional network (VAN), and the salience network (SN) were selected by visual inspection and confirmed using the template-matching procedure. The template was provided in GIFT software (the RSN template), and the map of each component was spatially correlated with a specific network template. The component with the largest spatial correlation coefficients with each of these templates was chosen and reconfirmed by visual inspection. Finally, we identified 12 networks for subsequent analysis (the detailed descriptions are presented in the Results section, and the operation process is shown in Figure 1) [29].

### 2.7. Inter-Network Connectivity Analysis

Temporal correlations between different resting-state networks (RSNs) were calculated to investigate the effects of rTMS and neurotransmitter drug interventions on brain functional networks. The time course of each RSN was extracted from the ICA procedure, and the time courses of each pair of the 12 RSNs were used to calculate the functional network connectivity (FNC) and normalized with the Fisher r-to-z transformation. A two-sample *t*-test was employed to compare group differences in the FNCs at an uncorrected significance level of *p* < 0.05 [2,26].

### 2.8. Intra-Network Connectivity Analysis

First, a single-sample *t*-test (*p* < 0.05, FWE-corrected) was used in the two groups to create a sample-specific component map and a network mask. A two-sample *t*-test was then conducted to compare the intra-network difference between the two groups for each component within the corresponding network mask. The statistical threshold was as follows: voxel level, *p* = 0.005; cluster level, *p* < 0.05 (FWE-corrected), and cluster size >10 voxels. Therefore, a t-value map with a significant between-group difference was obtained, and the results were displayed using xjview software [15,26].

### 2.9. Statistical Analysis

We used SPSS software (IBM SPSS Statistics 26) for data statistics, and the measurement data conforming to the normal distribution were expressed as the mean ± standard deviation, and the non-normal distribution data were expressed as the median in (lower quartile, upper quartile). The measurement data between the two groups were normalized using a stand-alone sample *t*-test and the non-normally distributed data were tested using Mann–Whitney U. The Spearman rank correlation analysis between brain functional characteristics and cognitive performance was further performed. The thresholds were set at *p* < 0.05.

## 3. Results

### 3.1. Demographic and Neuropsychological Data

In this study, four subjects were excluded from the analysis due to excessive head movement (*n* = 4). In addition, two subjects were not included in the cognition analysis due to incomplete cognitive scale data (*n* = 2). Ultimately, 30 subjects were included in the imaging analysis, and 28 subjects from the rTMS group were included in the cognition analysis (Figure 1 and Table 1). As illustrated in Table 1, the study enrolled 28 patients with cognitive impairment (11 males and 17 females), and the demographic data indicated no significant between-group differences in age, sex, educational level, MoCA scores, information processing speed, language, or visuospatial processing function (*p* > 0.05). The rTMS-alone intervention group exhibited significantly lower scores in general cognition (MMSE, *p* = 0.002), episodic memory (*p* = 0.022), and executive function (*p* = 0.002) than the combined intervention group.

### 3.2. ICA and Determination of RSNs

**Figure 2:**
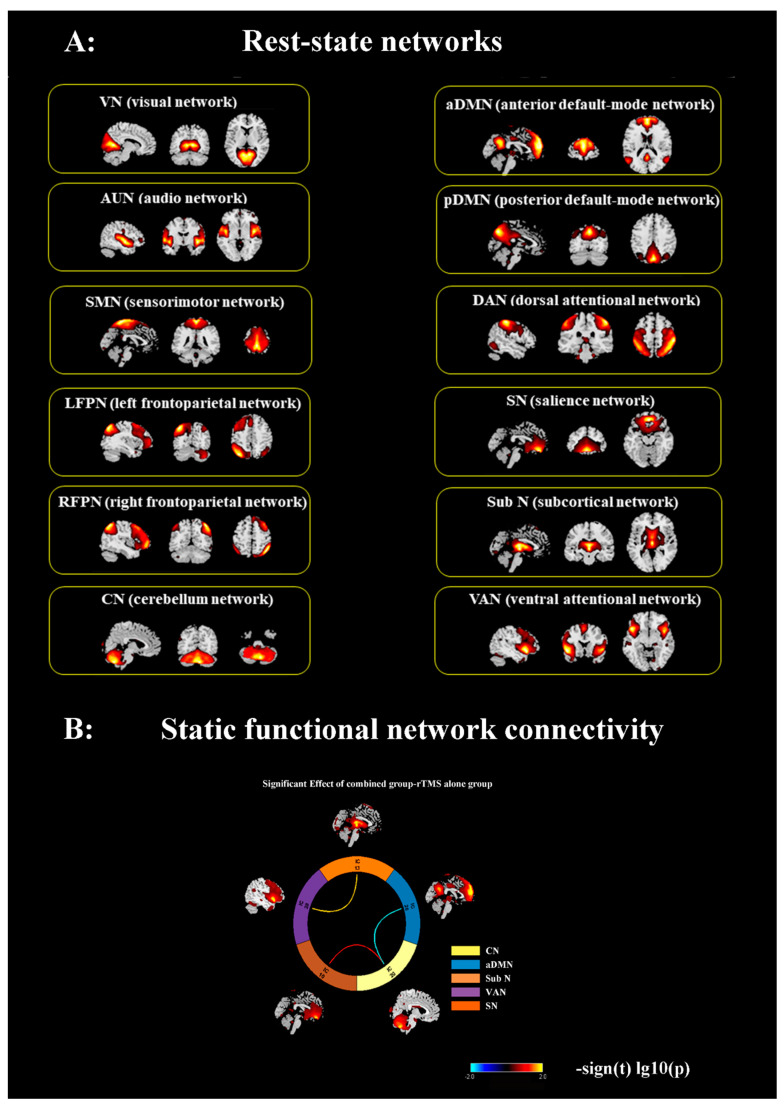
(**A**) Spatial maps of 12 selected RSNs. (**B**) sFNC is estimated as the pairwise correlation of the time courses. The circle plot shows differences in inter-network functional connectivity between two groups. Color of the lines connecting the networks is scaled to the color bar. Abbreviations: RSNs: resting-state networks; sFNC, static functional network connectivity.

Out of 34 components, we screened 12 networks that were in line with the previous studies [25,28,30,31,32]). The left and right frontoparietal network (LFPN, RFPN) consists primarily of the dorsolateral prefrontal cortex (DLPFC) and posterior parietal cortex (PPC). The anterior default mode network (aDMN) mainly involves the medial prefrontal (mPFC) and anterior cingulate cortex (ACC). The posterior DMN (pDMN) primarily includes the posterior cingulate cortex (PCC)/precuneus (Pcu) and the bilateral lateral parietal cortex. The sensorimotor network (SMN) mainly includes the bilateral precentral gyrus (PreCG) and postcentral gyrus (PostCG). The salience network (SN) mainly includes the dorsal ACC (dACC), bilateral VLPFC, and anterior insula (AI). The dorsal attention network (DAN) includes the interparietal sulcus and the junction of the precentral superior frontal sulcus bilaterally. Core regions of the ventral networks (VANs) are the inferior parietal lobule (IPL) and the adjacent temporo-parietal junction (TPJ). The visual network (VN) consists of the bilateral occipital lobe (OL). The auditory network (AN) primarily includes the bilateral superior temporal gyrus (STG) [2,27,33,34,35,36,37,38] (as shown in Figure 2).

### 3.3. Combined Intervention Reconstruct Inter-Network Connectivity of Cerebellum

**Figure 3:**
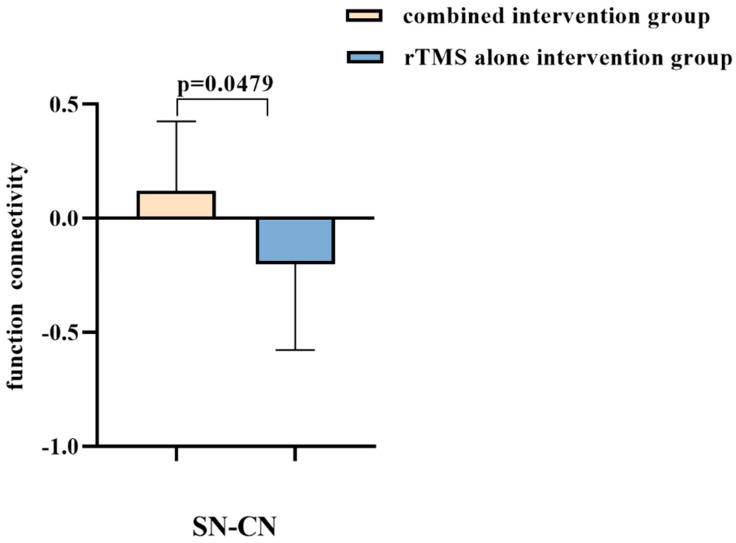
Inter-network connectivity differences between the rTMS-alone intervention group and the combined intervention group. Abbreviations: CN: cerebellum network; SN: salience network.

In comparison to the rTMS-alone intervention group, the combined intervention group demonstrated functional network connectivity (FNC) enhancement, including increased connectivity of CN-SN and subN-VAN (*p* < 0.05, uncorrected). Additionally, significantly reduced inter-network connectivity was observed in the CN and aDMN (*p* < 0.05, uncorrected). Figure 3 and Table 2 depict between-group differences in inter-network connectivity.

### 3.4. Combined Intervention Improves the Strength of Intra-Network Connectivity within Frontal-Parietal Regions

**Figure 4:**
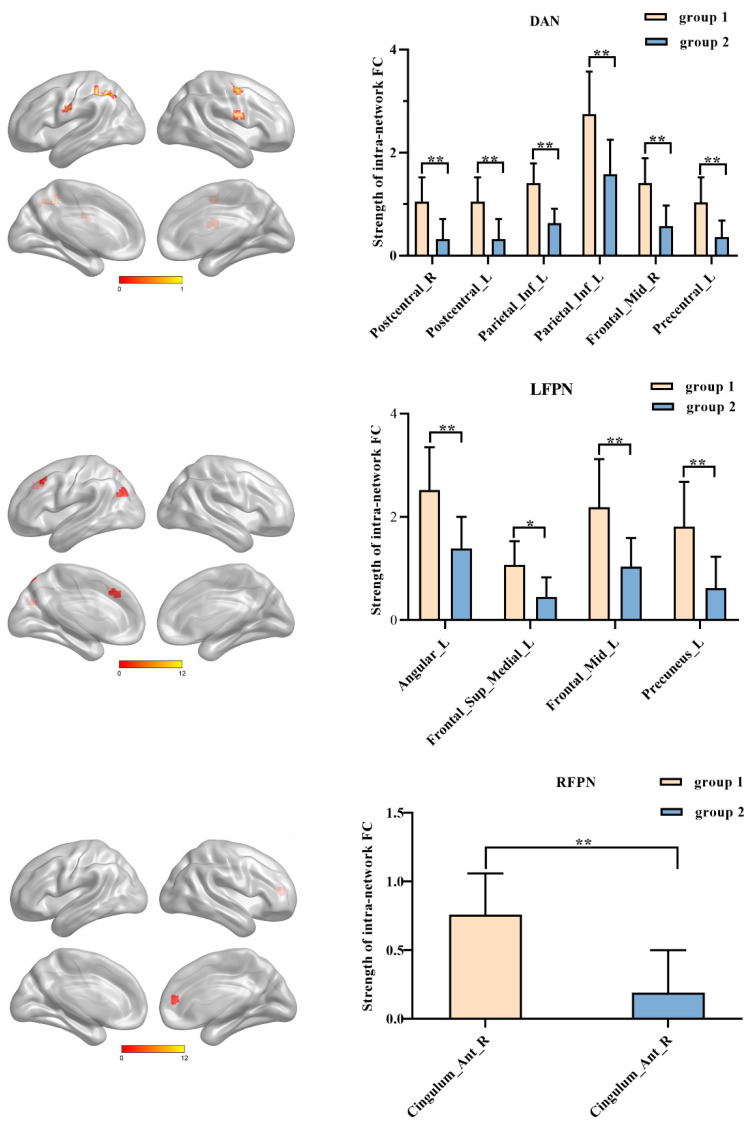
Differences in the intra-network functional connectivity (FC) analysis. The y-axis represents the Z-values of the mean value of intra-network FC strength, the x-axis represents the brain regions of the resting-state networks (RSNs). Error bars indicate the standard errors of the means (* *p* < 0.005, uncorrected; ** *p* < 0.0001, uncorrected), group 1: the Combined intervention; group 2: the rTMS-alone intervention.

The combined intervention group exhibited significantly increased intra-network connectivity when compared to the rTMS-alone intervention group, primarily involving frontal-parietal regions (Figure 4 and Table 3, *p* < 0.005, uncorrected). Specifically, (i) functional connectivity of Frontal_Sup_Medial_L/Angular_L/Frontal_Mid_L/Precuneus_L of LFPN exhibited significant increases. (ii) Significant increases were observed in Cingulum_Ant_R of RFPN. (iii) Connectivity improvements in Postcentral_L/Postcentral_R/Parietal_Inf_L/Frontal_Mid_R/Precentral_L were associated with DAN.

### 3.5. Differential Inter-Network/Intra-Network Connectivity Patterns and Behavioral Significance

**Figure 5:**
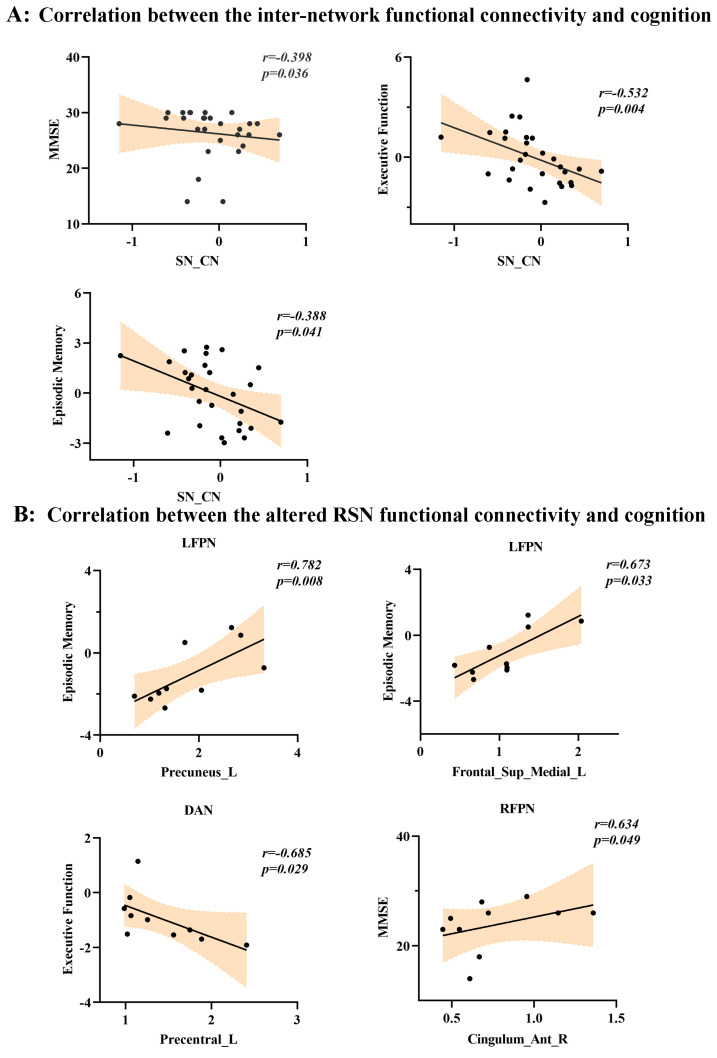
(**A**) The correlation between inter-network functional connectivity and clinical assessments. The y-axis represents different cognitive domains, while the x-axis represents the Z-values of inter-network functional connectivity. In addition, (**B**) shows the correlation between the altered resting-state network (RSN) functional connectivity and clinical assessments. The y-axis represents different cognitive domains, while the x-axis represents the Z-values of intra-network functional connectivity (FC).

Inter-Network connectivity: The Spearman rank correlation analysis revealed that the connectivity between CN and SN was correlated with multiple cognitive domains, including general cognition (i.e., MMSE) (r = −0.398, *p* = 0.036), episodic memory (r = −0.388, *p* = 0.041), and executive function (r = −0.532, *p* = 0.004) (Figure 5A).

Intra-network connectivity: this study determined correlations between the 12 RSNs and conducted cognitive assessments, and found that increased functional connectivity within the LFPN involving the left angular gyrus (r = 0.697, *p* = 0.025) and left precuneus (r = 0.782, *p* = 0.008) was positively correlated with episodic memory. Decreased functional connectivity of the left precentral gyrus within the DAN was negatively associated with executive function (r = −0.685, *p* = 0.029). Additionally, increased functional connectivity of the anterior cingulate and paracingulate gyri within RFPN was positively correlated with MMSE scores (Figure 5B).

## 4. Discussion

This study is the first to explore the effects of combining neurotransmitter drugs with rTMS intervention on brain network in patients with cognitive impairment. We compared the differences in the 12 RSNs between the combined intervention group and rTMS-alone intervention group, and found that the combined intervention improved the inter-network connectivity of the cerebellum and enhanced the strength of intra-network connectivity within frontal-parietal regions, which was closely related to cognitive improvement.

### 4.1. Neurotransmitter Drugs Combined with rTMS Intervention Can Reconstruct Functional Connectivity Associated with Cerebellum

The cerebellum has been known to play an important role in motor control [38,39]. There is growing evidence that the cerebellum is involved not only in motor function, but also in sensory and cognitive processes [40]. The cerebellum is a complex structural and functional region that is functionally connected to multiple brain networks and is involved in processes such as cognitive, emotional, and sensorimotor processes [41]. For example, Ferrari et al. demonstrated that the cerebellum plays a role in short-term memory for the order of incoming visual stimuli [40]. In addition, Gatti’s research shows that the right cerebellum has a causal relationship with words related to integrative semantics [42]. Therefore, previous studies have suggested that the cerebellum was closely associated with cognitive function [30,43,44,45,46].

Studies have shown that in patients with MCI and AD, there is a significant disruption of cortico-cerebellar functional connectivity, particularly in regions of the default mode network (DMN) and frontoparietal network (FPN). The DMN is a brain network that includes the medial prefrontal cortex, posterior cingulate cortex, and bilateral parietal cortex, and it is involved in various cognitive processes such as self-referential thinking, mind-wandering, and memory. It is considered the most vulnerable brain network in AD, and a reduced connection of the cerebellar network to the DMN in AD patients may be a contributing factor to cognitive impairment [47].

The present study found that the combined intervention group had enhanced connectivity between CN and SN, while decreasing connectivity between CN and aDMN when compared to the rTMS-alone intervention group. The functional connectivity between CN and SN was also found to be correlated with MMSE scores and episodic memory. Our results were consistent with those previous studies. For example, a large number of studies have shown that rTMS intervention can affect cerebellar function, including motor and cognitive domains. In the motor field, rTMS can affect cerebellum visual guidance, motor surround inhibition, motor adaptation, and learning. In the cognitive domain, rTMS can influence the cognitive processes involved in the cerebellum, including verbal working memory, semantic association, and predictive language processing [48]. It is important to acknowledge that while rTMS has a direct effect on neurons in the stimulated area, it may also indirectly modulate neural responses in other nearby or distant regions, such as the motor cortex or prefrontal cortex [40,49,50]. Transcranial magnetic stimulation (TMS) is known to directly modulate inhibitory activity of Purkinje cells located in the cerebellar cortex, which in turn affects the activity of the cerebral cortex through the thalamus. As a result, rTMS not only targets the intended brain region but can also indirectly modulate neural responses in other proximal or distal regions, such as the motor cortex or prefrontal cortex. Therefore, the effects of rTMS on brain function and behavior may extend beyond the targeted area and could potentially result in functional changes in other brain regions [51,52]. The results suggest that rTMS intervention targeting the left angular gyrus in this study may affect cognitive function by affecting the functional connection strength between the cerebellar cortex and the cerebral cortex.

In addition, much of the previous research on TMS affecting cerebellar function has focused on semantic memory [42]. The results of our study also proved this point. In this study, the FC between CN and SN was significantly enhanced in the combined intervention group, which was related to language function. Moreover, we also found that FC enhancement between CN and SN in patients in the combined intervention group was also related to MMSE scores and executive function. Summing up the above, we suspect that rTMS may affect cognitive function by inducing changes in the functional connectivity between the cerebellum and the cortex, regulating cerebellar excitability. Previous studies of drugs such as donepezil and memantine have not reported on the effects of neurotransmitters on functional connections between cerebellar networks and other networks, so we speculate that this may be a new finding regarding rTMS treatment, but the underlying mechanism needs more evidence to be proven.

### 4.2. Neurotransmitter Drugs Combined with rTMS Intervention Can Enhance Functional Connectivity within Frontal-Parietal Regions

An experiment utilizing repetitive transcranial magnetic stimulation (rTMS) to target the left angular gyrus has demonstrated the crucial role of this brain region in scenario simulation and memory [53], with the left angular gyrus situated at the convergence of brain regions supporting various cognitive processes, such as language, attention, and semantic, numerical, and social cognition [54,55]. The angular gyrus, as a node of the DMN, exhibits connections with the frontoparietal control network, which plays a role in executive control processes during cognitive tasks [56]. Additionally, the angular gyrus exhibits connections with the precuneus and the mid-cingulate cortex, which are believed to play a role in mediating various aspects of memory function, including memory retrieval tasks [57]. Previous studies have shown that rTMS targeting the left angular gyrus may impact other core regions, such as the hippocampus and precuneus, which are associated with visual memory and posterior cingulate/precuneus activation [53]. In particular, the activation of visual memory is closely related to memory processing (hippocampus and parahippocampal cortex) and posterior cingulate/precuneus [58,59,60,61]. Similar studies have suggested that the application of rTMS can affect episodic memory performance by targeting specific lateral parietal regions that are connected to the hippocampus [13,62].

The fronto-parietal network included the bilateral DLPFC (middle frontal gyrus) and parietal (superior parietal gyrus) cortex, which play a crucial role in the top-down control of attention and in modulating cognition in AD. Prior studies have indicated that cognitive control is executed through the adaptable reorganization of the FPN in relation to various cognitive domains [63,64,65,66].

We found that the FC of the left precuneus and Frontal_Sup_Medial_L, which belong to LFPN, were improved significantly in the combined intervention group compared to in the rTMS-alone intervention group. The subsequent related analyses confirmed that the Z-values of the left precuneus and Frontal_Sup_Medial_L were positively correlated with the episodic memory. We suggest that this improvement was supported by changes in cortical activity of the precuneus and Frontal_Sup_Medial_L and their connectivity with the frontal-parietal network. Our findings are in accordance with previous studies. Giacomo et al. found a significant beneficial effect of rTMS intervention that targeted the precuneus in improving episodic memory [67].

Prior studies have demonstrated that the dorsal attention network (DAN) consists of both cortical and cerebellar nodes that are associated with attentional control [68]. Recent research has found that increased functional connectivity (FC) within the DAN and enhanced connections between the cerebellum and cortical network nodes were strongly correlated with increased activation during multiple attention tasks, indicating a significant relationship between connectivity and cognitive performance [50]. This finding is consistent with our previous discussion that rTMS can rebuild the functional connection between the cerebellum and cortex.

Furthermore, our findings indicate that the functional connectivity (FC) of the Precentral_L node within the dorsal attention network (DAN) was greater in the combined intervention group than in the rTMS-only intervention group. Additional analyses revealed that the Z-values of the Precentral_L node were negatively associated with executive functions. In addition, the heightened connectivity within the DAN in the combined intervention group is related to poorer cognitive function, potentially indicating a compensatory mechanism. This mechanism proposes that hyperactive brain regions increase their activity to compensate for cognitive decline in other brain regions to preserve cognitive function [69].

The rTMS intervention in this study targeted the left hippocampus, and all participants received four weeks of treatment with rTMS aimed at the left angular gyrus. The results demonstrated enhanced functional connectivity (FC) of the precuneus, which was associated with episodic memory and MMSE scores, consistent with previous findings. Notably, neurotransmitters can have similar effects, as evidenced by a previous study which found that just five days of galantamine treatment in MCI patients resulted in increased activation in the left hippocampus, prefrontal, anterior cingulate, and occipital cortices during face encoding, as well as increased activity in the right precuneus and middle frontal gyrus during working memory tasks [70]. A previous study has reported that the ventral default mode network (DMN) comprises a collection of regions spanning from the precuneus and posterior cingulate cortex to the parahippocampal region via the retrosplenial cortex [71]. They also found that three months of donepezil treatment significantly enhanced functional connectivity within the network, including the para-hippocampal gyrus [72]. Moreover, previous studies have shown that donepezil can improve cognitive function in patients with Alzheimer’s disease (AD) through modulation of spontaneous brain activity. Specifically, improvements in cognitive function have been associated with increased spontaneous activity in the right gyrus rectus, precentral gyrus, and left superior temporal gyrus [73].

The results suggest that the functional connection of the precuneus and frontal lobe is enhanced, and positive correlation with episodic memory and cognition may be a synergistic effect between rTMS and neurotransmitters.

Previous research has also shown that upregulating frontal systems activation can improve cognitive performance [6,74,75]. Thus, the enhancement of precentral gyrus functional connection in the combined intervention group may be due to the action of neurotransmitters.

Overall, the results indicate that the combined intervention is more effective than rTMS alone in enhancing functional connectivity within FPN and DMN, which can improve cognitive function in patients with cognitive impairment.

### 4.3. Limitations and Prospects

There were several limitations in our study. First, many imaging studies have demonstrated that neurotransmitters activate the brain, but it is still unclear whether the changes in brain activation caused by these drugs are associated with changes in cognitive outcomes, and whether these associations have significant cognitive benefits. In addition, due to sample size limitations, we chose a less stringent threshold for voxels and clumps, and the changes in brain functional network connectivity between groups were not strictly corrected. Second, our study was a short-term study; there was no long-term follow-up of patients and, due to conditional restrictions, we failed to include a group of patients who had not been treated with rTMS but were taking neurotransmitters as a control group. In future studies we will include control groups and expand sample sizes to draw stronger conclusions. In addition, we will conduct long-term follow-up of subjects to explore the long-term effects of drug and rTMS interventions.

## 5. Conclusions

In summary, the present findings clearly demonstrated that co-intervention of neurotransmitter drugs and rTMS showed potential for cognitive improvement via the reconstructed inter-network connectivity of the cerebellum and the enhanced intra-network connectivity of frontal-parietal regions, although additional studies will be needed to shed light on more fine-grained questions regarding the effect of the combined intervention.

## Figures and Tables

**Table 1:** Demographic and neuropsychological data.

Items	Combined Intervention Group	rTMS-Alone Intervention Group	*p* Value
	(*n* = 10)	(*n* = 18)	
**Demographics**			
Age (years) means ± SD	68.40 ± 4.95	65.78 ± 8.30	0.371
Education (years) IQR	9.00 (12.75−9.00)	12.00 (15.00−9.00)	0.146
Gender (male/female)	4/6	7/11	0.954
**General cognition**			
MMSE	25.50 (26.50−21.75)	29.00 (30.00−27.00)	0.002 *
MoCA	21.00 (26.25−18.75)	24.50 (26.00−22.50)	0.332
**Composition Z scores of each cognitive domain**			
Episodic Memory	−1.07 ± 1.43	0.59 ± 3.512	0.022 *
AVLT-DR	3.80 ± 2.49	6.06 ± 3.73	0.100
VR-DR (WMS)	−0.91 (0.124−(−1.46))	0.73 (1.16−0.02)	0.010 *
Information Processing Speed	−1.34 (−0.28−(−2.61))	1.06 (2.26−(−1.76))	0.077
TMT-A (inverse)	−0.44 (−0.98−(−0.19))	0.07 (0.88−(−0.51))	0.010 *
Stroop A (inverse)	−0.33 ± 0.79	0.18 ± 1.08	0.201
Stroop B (inverse)	−0.42(−0.24−(−0.72))	0.33 (0.72−(−0.48))	0.035 *
Language	−0.46(0.67−(−1.20))	0.57 (1.35−(−1.32))	0.408
CVF	−0.31 ± 0.76	0.17 ± 1.09	0.224
BNT	0.33(0.55−(−0.11))	0.24 (0.52(−0.30))	0.689
Visuospatial Processing Function	0.39(0.93−(−0.97))	0.93 (0.93−(−0.16))	0.460
CDT	0.62(0.62−(−0.74))	0.62 (0.62−(−0.74))	0.906
VR-C	0.31(0.31−0.20)	0.31(0.31−0.31)	0.724
Executive Function	−1.17 (−0.48−(−1.52))	0.55 (1.49−(−0.75))	0.014 *
TMT-B (inverse)	−0.44 (−0.59−(−0.31))	−0.18 (0.51−(−0.56))	0.270
Stroop C (inverse)	−0.77 (0.075−(−1.20))	0.44 (0.96−(−0.31))	0.004 *

Values are presented as the mean ± standard deviation (SD). The *p*-value was obtained by two-sample *t*-test. * indicates statistically significant differences in data between the two groups of subjects after receiving the same rTMS intervention, *p* < 0.05. Abbreviations: MMSE, mini mental state examination; MoCA-BJ, Beijing version of the Montreal Cognitive Assessment; AVLT-DR, Auditory Verbal Learning Test-delayed recall; VR-DR, visual reproduction-delay recall; CDT, Clock Drawing Test; VR-C, visual reproduction-copy; CVF, category verbal fluency; BNT, Boston Naming Test; TMT-A and TMT-B, Trail Making Test-A and B; Stroop A, B and C, Stroop Color and Word Tests A, B, and C.

**Table 2:** Differences in functional network connections between groups.

Inter-Network FC	*t*-Value	*p*-Value
aDMN-CN	−2.81	0.009
SN-CN	2.07	0.0479
sub N-VAN	2.57	0.0158

**Table 3:** Brain regions showing significantly different functional connectivity within RSNs between groups.

	Brain Regions	Cluster Size	Peak Intensity	Peak MNI Coordinate
		(mm^3^)		x,y,z (mm)
**LFPN**				
	Angular_L	459	4.0923	−45 −75 30
	Frontal_Sup_Medial_L	540	3.5118	−9 33 36
	Frontal_Mid_L	459	3.9411	−39 24 45
	Precuneus_L	270	4.3838	−12 −72 57
**aDMN**				
	Frontal_Sup_Medial_L	378	3.8797	−3 57 21
	Frontal_Mid_L	648	4.0521	−27 33 30
	Frontal_Sup_Medial_R	432	4.2036	12 45 45
	Precentral_L	432	4.1363	−39 −9 57
**CN**				
	Fusiform_L	378	3.8623	−33 −48 −21
**DAN**				
	Postcentral_R	405	3.5456	63 3 21
	Postcentral_L	324	4.3801	−57 −6 30
	Parietal_Inf_L	891	4.5272	−39 −57 42
	Parietal_Inf_L	540	4.4364	−45 −45 51
	Frontal_Mid_R	1026	4.754	39 −6 51
	Precentral_L	270	4.6901	−36 −12 51
**pDMN**				
	Caudate_L	351	4.3674	−9 −3 3
	Precuneus_L	378	4.6397	−6 −51 48
	Precuneus_R	351	3.3809	12 −51 51
**RFPN**				
	Cingulum_Ant_R	432	4.9952	12 48 12
**SMN**				
	Cingulum_Mid_R	621	3.6626	9 −36 45
	Precuneus_L	270	3.6859	−15 −45 51
	Postcentral_L	1647	4.9895	−21 −30 63
	Precuneus_R	945	4.4568	9 −63 60
**VAN**				
	Frontal_Inf_Orb_L	378	4.5632	−27 15 −18
	Putamen_L	405	4.6378	−12 6 −3
	Insula_R	270	3.9312	42 0 0
	Frontal_Mid_L	297	3.92	−48 27 39
	Supp_Motor_Area_L	540	4.2135	−3 3 48

## Data Availability

The data used to support the findings of this study are available from the corresponding author upon request.
